# Defunctioning stoma before neoadjuvant treatment or resection of endoscopically obstructing rectal cancer

**DOI:** 10.1007/s00384-023-04318-8

**Published:** 2023-01-26

**Authors:** Gustav Sandén, Johan Svensson, Ingrid Ljuslinder, Martin Rutegård

**Affiliations:** 1 Department of Surgical and Perioperative Sciences, Surgery Umeå University, 901 85 Umeå, Sweden; 2 Department of Statistics, Umeå School of Business and Economics, Umeå University, Umeå, Sweden; 3 Department of Radiation Sciences, Oncology, Umeå University, Umeå, Sweden; 4 Wallenberg Centre for Molecular Medicine, Umeå University, Umeå, Sweden

**Keywords:** Rectal Cancer, Endoscopy, Bowel Obstruction, Stoma

## Abstract

**Aim:**

To investigate whether patients with endoscopically untraversable rectal cancer may benefit from a defunctioning stoma created before neoadjuvant therapy or resectional surgery.

**Methods:**

This retrospective study comprise patients diagnosed with rectal cancer during 2007–2020 in Region Västerbotten, Sweden. The primary outcome was time between diagnosis and any treatment, while survival and the incidence of complications were secondary outcomes. Excluded were patients without endoscopic obstruction, patients already having a stoma, patients with recurrent disease, palliative patients, and patients receiving a stoma shortly after diagnosis due to any urgent bowel-related complication. Data were obtained from the Swedish Colorectal Cancer Registry and medical records. Kaplan–Meier failure curves were drawn, and a multivariable Cox regression model was employed for confounding adjustment.

**Results:**

Out of 843 patients, 57 remained after applying exclusion criteria. Some 12/57 (21%) patients received a planned stoma before treatment, and the remainder received upfront neoadjuvant therapy or surgery. Median time to any treatment was 51 days for the planned stoma group and 36 days for the control group, with an adjusted hazard ratio of 0.28 (95% confidence interval: 0.12–0.64). Complications occurred at a rate of 5/12 (42%) and 7/45 (16%) in the planned stoma group and control group, respectively. Survival was similar between groups.

**Conclusion:**

A planned stoma results in treatment delay, but it remains unclear whether this is clinically relevant. Complications were more common in the planned stoma group, although the data are limited. While larger studies are needed, it seems feasible to avoid defunctioning stomas even in endoscopically obstructing rectal cancers.

**Supplementary Information:**

The online version contains supplementary material available at 10.1007/s00384-023-04318-8.

## Introduction

Approximately 2100 people are diagnosed with rectal cancer every year in Sweden. The treatment typically consists of surgery with resection of the tumour and surrounding tissue. In addition, neoadjuvant and/or adjuvant radiation therapy or chemotherapy may also be used [1, 2]. Sometimes the tumour grows in a way that partially or completely obstructs the bowel, making it difficult or impossible to perform a complete colonoscopy, referred to as an endoscopically obstructing tumour. These patients may show no clinical signs of obstruction, but are at risk of progressing into, for example, large bowel obstruction with subsequent bowel perforation. To avoid this, a defunctioning stoma is sometimes created before the initiation of neoadjuvant oncological treatment or resectional surgery [3]. The different types of stomas can be divided into ileostomy, transverse colostomy, and sigmoidostomy. Creating an ileostomy is simple from a technical point of view, but can sometimes cause postoperative dehydration and renal failure [4]. Transverse colostomies are associated with stoma prolapse and usually require a more extensive dissection during surgery, but are otherwise a valid option [5]. Sigmoidostomies are regarded by some surgeons as suboptimal considering the proximity to the tumour, as such a stoma could cause interference during future resection attempts. The rationale for creating a defunctioning stoma in complete large bowel obstruction is obvious, whereas doing so before neoadjuvant therapy or resectional surgery due to incomplete obstruction has been studied less; it is possible that defunctioning stomas are created to a higher degree than necessary for these patients, causing morbidity that otherwise could have been avoided [6]. There are some reports on the impact of defunctioning stomas for patients with endoscopically obstructing rectal tumours, but these studies are not population-based, with associated problems of selection bias [3]. Our aim was to investigate the potential merits and drawbacks of defunctioning stomas before initiation of any treatment in patients with endoscopically obstructing rectal cancer in Region Västerbotten, Sweden.

## Methods

### Study design

In this retrospective study, patients diagnosed with rectal cancer from January 2007 to December 2020 were identified using the Northern Regional Cancer Centre and the Swedish Colorectal Cancer Registry. Patients from Region Västerbotten, Sweden, were included. The Colorectal Cancer Registry and medical records were used to extract relevant clinical and demographic data.

### Chart review

The endoscopy examinations for each patient were scrutinized to identify endoscopically obstructing rectal tumours. Once identified, these patients were reviewed in further detail regarding scope type (only the thinnest scope type used was noted), curative or palliative intent of offered treatment, clinical signs of obstruction (or other indications for an emergency stoma), pretreatment stoma type, starting date of neoadjuvant therapy, and complications occurring between diagnosis and resectional surgery. Complications were assessed according to the extended Clavien-Dindo classification of surgical complications, although only complications classified as grade II or above were considered in this study [7]. Moreover, only the highest classified complication for each patient was assessed. Patients excluded were those not matching the target demographic with regard to region and diagnosis, patients with missing or inadequate medical records describing the endoscopy examination, patients with a stoma prior to diagnosis, patients with recurrent rectal cancer, patients deemed to be palliative shortly after diagnosis, and patients receiving an emergency stoma shortly after diagnosis due to any urgent bowel-related complication. Patients with metastatic disease not initially deemed to be palliative were included. Patients who did not receive any neoadjuvant therapy before surgery were also included, as there may be situations where a pretreatment stoma could benefit such patients (e.g. delay to surgery due to patient comorbidity or logistical factors, while the tumour itself does not mandate oncological treatment).

The following symptoms were taken into consideration when assessing clinical signs of obstruction: abdominal pain, abdominal distension, vomiting, and ceased passage of stool. Patients were deemed to be palliative if assessed as such during the early multidisciplinary conferences following diagnosis, or if it was otherwise obvious that no curative treatment was intended. The neoadjuvant oncological treatment, if given, differed depending on the type of regime currently in use at the time of diagnosis. During the earlier part of the study period, a long-course radiation regime of 50.4 Gy with concomitant chemotherapy was common practice. This was, however, widely replaced by a short-course radiation regime consisting of 5 × 5 Gy followed by 4–6 courses of 5-fluourouracil-based chemotherapy during the latter part of the study period. Throughout the study period, short-course preoperative radiation therapy of 5 × 5 Gy without subsequent chemotherapy was also a standard option.

### Registry data

The information from the patients’ medical records was combined with data from the Colorectal Cancer Registry, which includes age, sex, date of diagnosis, date of resectional surgery, date of death, surgical technique, type of neoadjuvant therapy given, perioperative complications, tumour stage, and comorbidity. Tumour stage was classified using the tumour-node-metastasis staging system, while comorbidity was graded according to the American Society of Anaesthesiologists (ASA) classification [8, 9].

### Exposure and outcomes

The exposure was defined as receiving a planned defunctioning stoma before initiation of any treatment. Clinical and demographic characteristics were then tabulated and stratified by the presence of such a stoma. The primary outcome was time from diagnosis to any treatment (neoadjuvant therapy or resectional surgery). The secondary outcomes were time from diagnosis to neoadjuvant therapy and resectional surgery, respectively. For descriptive purposes, we also compared the occurrence of complications between exposure groups, as well as survival.

### Statistical analysis

The median and 95% confidence intervals (CIs) were used to describe time-to-event across groups. Kaplan–Meier failure curves and the log-rank test were used to visualize and make inferences about the time-to-event. A multivariable Cox regression model was employed with a minimal set of covariates for the primary outcome only, comprising age (continuous), sex (male, or female), tumour stage (I–II, III, or IV), and neoadjuvant therapy (none, radiotherapy, or chemoradiotherapy) [10]. Fisher’s exact test was performed to evaluate differences in postoperative complications. A complete case analysis was conducted throughout. For all analyses, the statistical software STATA version 16.1 (StataCorp, Houston, TX, USA) was used.

## Results

### Patients

**Fig. 1:**
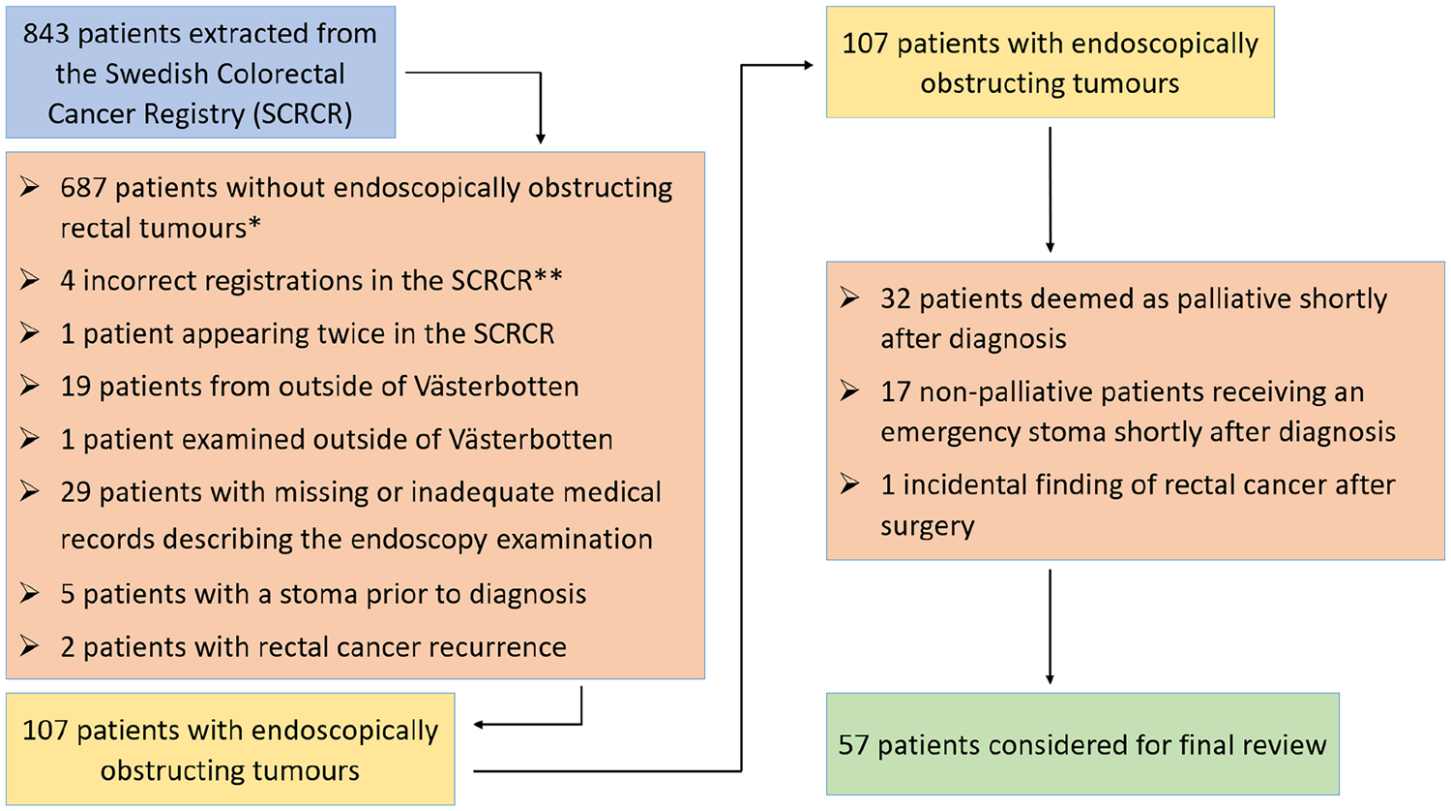
Study flowchart. Excluded patients are shown in brown boxes. Note that patients may fulfil multiple exclusion criteria. *Not including patients examined or occupied outside of Västerbotten, or patients with missing/inadequate medical journals describing the endoscopy examination. **2 cases of sigmoid cancer, 1 case of transverse colon cancer, 1 case of high-grade dysplasia

During the study period, 843 patients with rectal cancer were identified. After applying the exclusion criteria, 107 patients with endoscopically obstructing tumours remained. Of these, an additional 49 patients were excluded for being deemed as palliative (*n* = 32) and for receiving an emergency stoma (*n* = 17), respectively, shortly after diagnosis. Another patient was excluded for being diagnosed after an accidental finding of rectal cancer following surgery. In the end, 57 patients remained for analysis, as depicted by the study flowchart (Fig. 1). Out of these 57 patients, 12 received a planned defunctioning stoma before any treatment (planned stoma group); these stomas comprised five ileostomies, one transverse colostomy, and six sigmoidostomies. The remaining 45 patients received upfront neoadjuvant therapy or surgery (control group).

There were twice as many men than women in the planned stoma group, whereas the control group had a relatively even gender distribution. The ASA grade was similar between the two groups, with slightly more grade I patients in the planned stoma group and grade II patients in the control group. Moreover, the types of scopes used were also comparable across the groups. All 12 patients in the planned stoma group were intended for neoadjuvant therapy (although only 11 ultimately received this treatment), while a large minority of patients in the control group received upfront surgery. As for tumour stage, the planned stoma group had more advanced tumours overall; stage II tumours in the control group were almost as common as stage IV tumours in the planned stoma group. Seven patients, four (33.3%) in the planned stoma group and three (6.7%) in the control group, later progressed to a palliative state due to tumour progression. One of these seven patients received a non-curative resection, and one did not receive any curative treatment at all. For demographic and clinical details, see Table 1.

**Table 1:** Demographic and clinical details for 57 patients with an endoscopically obstructing rectal cancer, stratified by a planned pretreatment stoma

	**No stoma (*n* = 45)**	**Stoma (*n* = 12)**
***Categorical variables***	***N* (%)**	***N* (%)**
**Sex**		
Male	21 (46.7)	8 (66.7)
Female	24 (53.3)	4 (33.3)
**ASA fitness grade**		
I	3 (6.7)	3 (25.0)
II	27 (60.0)	3 (25.0)
III–IV	10 (22.2)	2 (16.7)
* Missing*	5 (11.1)	4 (33.3)
**Year of diagnosis**		
2007–2013	30 (66.7)	10 (83.3)
2014–2020	15 (33.3)	2 (16.7)
Scope type used*		
Colonoscope	34 (75.6)	9 (75.0)
Ultrathin colonoscope	2 (4.4)	0 (0.0)
Rigid sigmoidoscope	6 (13.3)	2 (16.7)
Gastroscope	3 (6.7)	1 (8.3)
**Clinical tumour stage (TNM)**		
I	2 (4.4)	0 (0.0)
II	18 (40.0)	2 (16.7)
III	8 (17.8)	4 (33.3)
IV	15 (33.3)	5 (41.7)
* Missing*	2 (4.4)	1 (8.3)
**Neoadjuvant therapy**		
None	18 (40.0)	1 (8.3)
Radiotherapy	17 (37.8)	2 (16.7)
Chemoradiotherapy	10 (22.2)**	9 (75.0)
**Type of resectional surgery**		
Anterior resection	26 (57.8)	3 (25.0)
Abdominoperineal excision	9 (20.0)	4 (33.3)
Hartmann’s procedure	7 (15.6)	2 (16.7)
No resection	3 (6.7)	3 (25.0)
**Perioperative complications during resection**		
No	34 (75.6)	8 (66.7)
Yes	11 (24.4)	4 (33.3)
***Continuous variables***	**Median (IQR)**	**Median (IQR)**
** Age (years)**	69 (62–79)	67.5 (52.5–71)
** Tumour height (cm)**	11 (8–13)	9 (8–11)

*ASA* American Society of Anaesthesiologists, *TNM* tumour-node-metastasis staging system, *IQR* interquartile range

*If several different types of scopes were used, only the thinnest scope was considered

**One of these patients received chemotherapy only

### Time to treatment

**Fig. 2:**
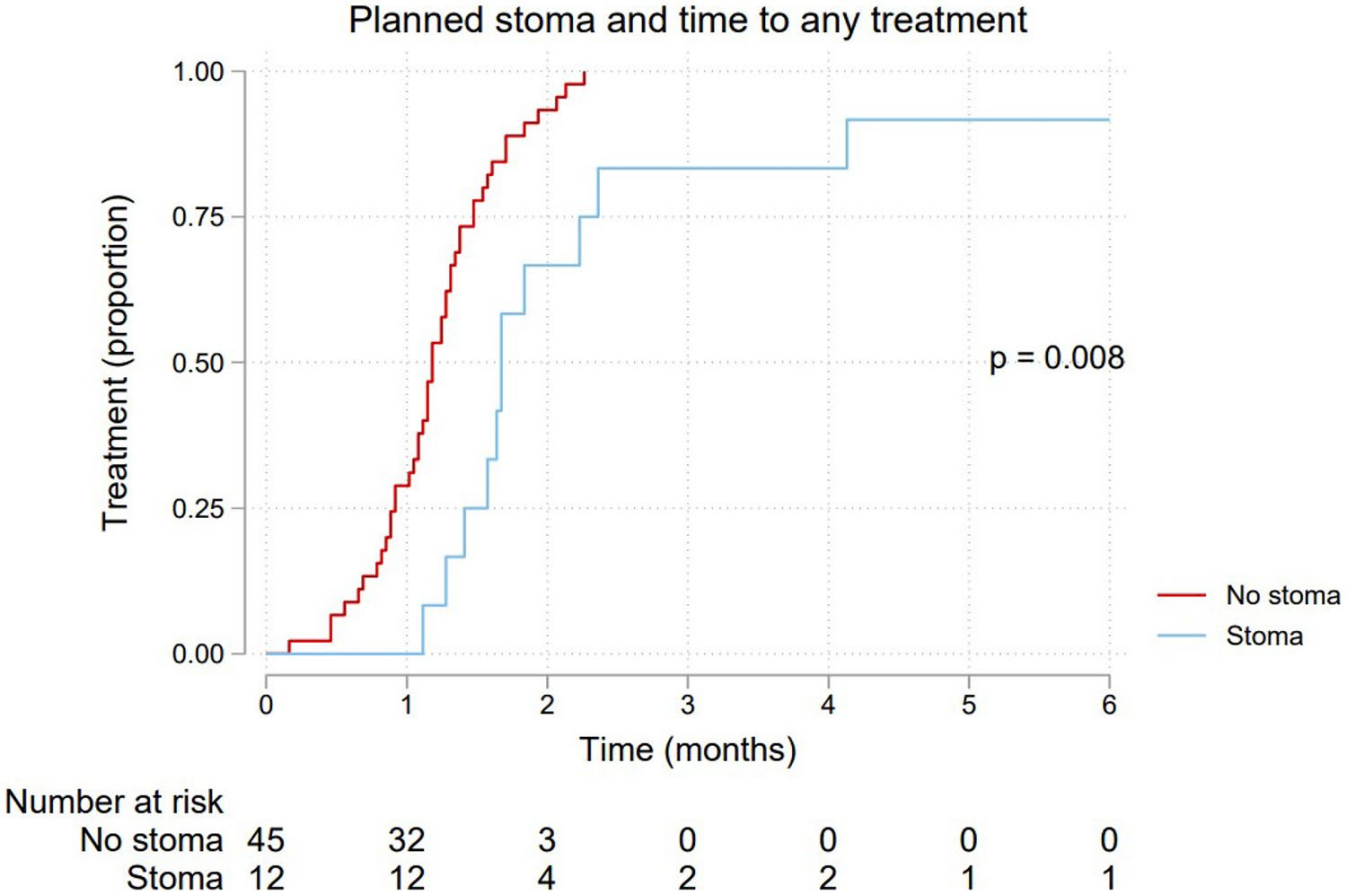
Kaplan–Meier failure curve showing time to any treatment for 57 patients with endoscopically obstructing rectal cancer, stratified by a planned pretreatment stoma

**Fig. 3:**
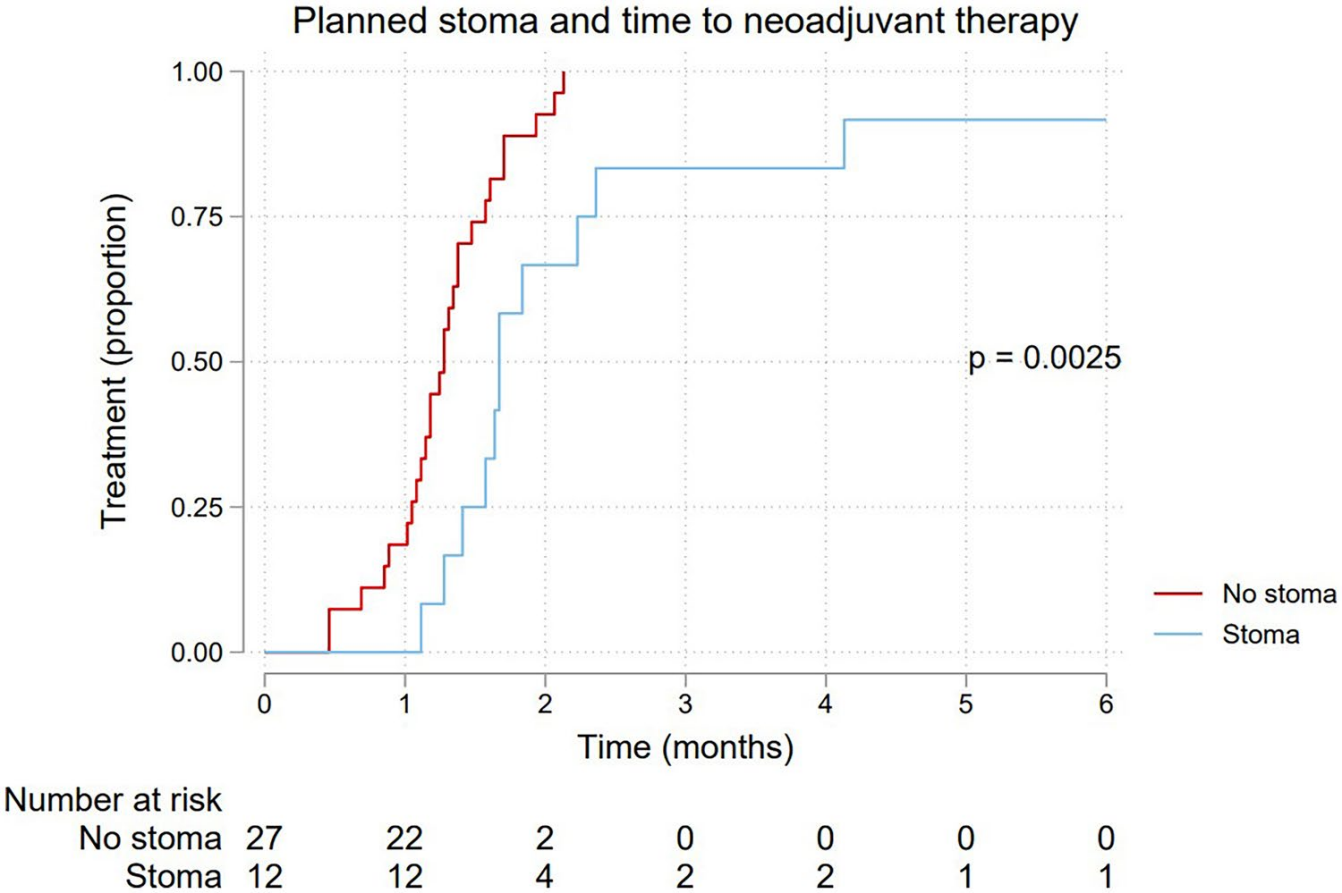
Kaplan–Meier failure curve showing time to neoadjuvant treatment for 39 patients with endoscopically obstructing rectal cancer, stratified by a planned pretreatment stoma

**Fig. 4:**
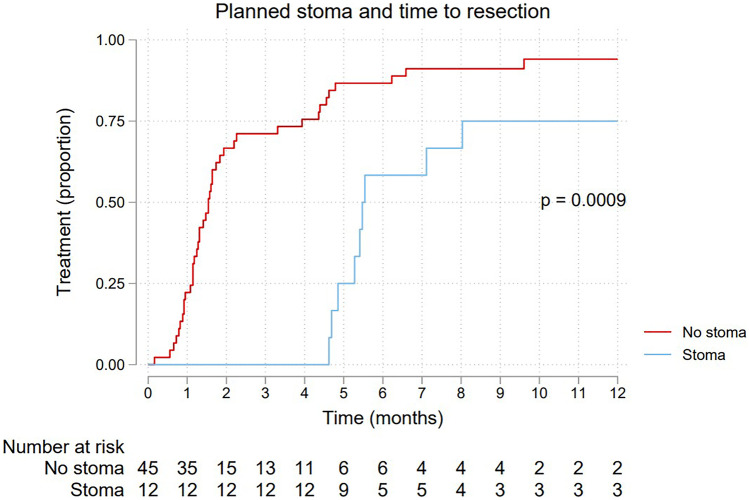
Kaplan–Meier failure curve showing time to resectional surgery for 57 patients with endoscopically obstructing rectal cancer, stratified by a planned pretreatment stoma

Median time to any treatment, neoadjuvant therapy and resectional surgery, is shown in Table 2. The differences in time between the groups are further depicted by the Kaplan–Meier failure curves shown in Figs. 2, 3, and 4, respectively. All three curves show a significantly longer time-to-event for the planned stoma group (*p* = 0.0080, 0.0025, 0.0009, respectively). After assessment at the first multidisciplinary conference(s), 18 patients were planned for upfront resectional surgery without any neoadjuvant treatment. These patients are excluded from the Kaplan–Meier failure curve shown in Fig. 3, depicting only patients planned for neoadjuvant therapy.

**Table 2:** Time to treatment for 57 patients with an endoscopically obstructing rectal cancer, stratified by a planned pretreatment stoma

***Time to treatment (days)***		**No stoma (*n* = 45)**	**Stoma (*n* = 12)**
	**Events/patients**	**Median (95% CI)**	**Median (95% CI)**
**Any treatment**	56/57	36 (33–39)	51 (39–72)
**Neoadjuvant therapy**	38/39	39 (33–42)	51 (39–72)
**Resection**	51/57	47 (38–56)	167 (143–NA)

*CI* confidence interval, *NA* not available

With Cox regression, the unadjusted hazard ratio (HR) was 0.30 with a 95% CI of 0.15–0.64. After adjustment for confounding, the adjusted HR was 0.28 (95% CI: 0.12–0.64), indicating an increased time to treatment in the planned stoma group.

### Complications

A total of 12 patients with complications classified as grade II or above according to the extended Clavien-Dindo classification of surgical complications were found across all 57 patients. All 12 patients had complications classified as either grade II or IIIb; no patient had a grade IIIa, IV, or V complication as their highest graded complication. In the planned stoma group, there were three patients (25.0%) with grade II complications and two patients (16.7%) with grade IIIb complications, resulting in a combined complication rate of 5/12 (41.7%). In the control group, the corresponding numbers of patients were four (8.9%) and three (6.7%), respectively, thus with a total complication rate of 7/45 (15.6%). However, the difference in complication rate was not statistically significant (*p* = 0.1036).

Both grade IIIb complications in the planned stoma group occurred following the stoma surgery. One patient needed reoperation due to bleeding following surgery, while the other patient developed an abscess and fistula in the abdominal wall adjacent to the stoma that required surgical intervention, including wound revision, abscess drainage, and skin grafting. The former patient started neoadjuvant therapy in a relatively timely manner (43 days after diagnosis), whereas the latter took considerably longer than usual to start (126 days after diagnosis).

In the control group, one patient sustained a stroke that required urgent carotid endarterectomy, while the other two patients with grade IIIb complications suffered a bowel perforation following malignant large bowel obstruction (281 and 293 days after diagnosis, respectively). The patient who had a stroke took longer than usual to start neoadjuvant therapy (63 days after diagnosis). The perforations, however, did not occur until after the patients were already deemed as palliative, and no delay in neoadjuvant therapy was seen in these cases (38 and 14 days after diagnosis, respectively). One of these patients ultimately had a non-curative tumour resection, while the other received an ileostomy. Regarding perioperative complications during resectional surgery, these occurred at a similar rate between the groups (Table 1).

### Survival

The median survival after diagnosis was 43 months (CI: 18–72) for the planned stoma group and 58 months (CI: 44–not estimable) for the control group. The survival difference between groups, however, was not significant (*p* = 0.4543). For details, see the Kaplan–Meier failure curve shown in Supp. Fig. S1.

## Discussion and conclusions

The results show a clear delay in time to treatment for the planned stoma group. This may not come as a surprise, considering the added procedure taking place before treatment for these patients. However, it is unclear whether this difference has any clinical relevance with regard to the oncological prognosis, as survival was similar in both groups. Evaluating the time-to-any-treatment and time-to-neoadjuvant-therapy categories, the majority of patients in the planned stoma group received treatment no more than a few weeks later than the average patient in the control group. The time-to-resection category is more difficult to assess, however, as the pronounced delay seen in this section is largely attributed to the long-course chemoradiotherapy regime more commonly used for patients in the planned stoma group. Nevertheless, further studies with a larger sample size and additional focus on prognosis are likely needed before these results can be applied to any clinical setting. In the meantime, one can only speculate that a timely initiation of treatment may be especially beneficial for patients with endoscopically obstructing tumours, as these tumours tend to be advanced and may require downstaging for local control [11].

As for complications, these were more common overall in the planned stoma group, albeit not statistically significantly so. Some of these complications were a direct consequence of the stoma surgery and likely could have been avoided had no stoma been constructed. Conversely, it is possible that the bowel perforations seen in the control group never would have happened with a defunctioning stoma already in place, although they also may have been a result of local disease progression. That being said, it is difficult to reach any firm conclusions given the limited data available, though it seems feasible to avoid defunctioning stomas in this situation, given the lack of higher complication rates in the control group.

The most common type of surgical technique used during resectional surgery varied between the groups, with more anterior resections in the control group. This could indicate that receiving a pretreatment stoma increases the risk of receiving a permanent colostomy following resectional surgery. However, it is important to note that the median tumour height is lower in the planned stoma group, predisposing for fewer anterior resections. Furthermore, some surgeons will prefer an abdominoperineal excision if a preoperative stoma already exists, especially if this is a loop sigmoidostomy, as this enables stoma division at the level of the abdominal wall and omits a need to create a novel stoma.

A similar study made by Patel et al. was published in 2012 [3]. The study had, after application of rather comparable exclusion criteria, a larger sample size of 85 patients. The patients in that study were also more homogenous regarding the choice of neoadjuvant treatments. However, the study was not population-based, with associated susceptibility of selection bias. Nonetheless, the authors concluded that the construction of a pretreatment stoma should only be advocated when symptoms of obstruction are present, as 90% of patients in the control group received their planned treatment without any prior major issues. Furthermore, they saw a significant delay in treatment initiation for the planned stoma group. A similar pattern can be seen in our population-based study, though such a small treatment delay may not be clinically significant.

There are limitations to our study. Despite including patients diagnosed during a relatively long period of 14 years, the final sample size consisted of no more than 57 patients. This makes it difficult to evaluate the results derived from this study and needs to be taken into consideration when reaching for any conclusions.

Apart from the small study population, there are other limitations as well. The process of determining which tumours are endoscopically obstructing and which ones are not relies on the type of scope used; different types of scopes vary significantly in both circumference and flexibility. Additionally, the skill and methodology of the endoscopist may also vary. Most of the 57 patients reviewed in this study were diagnosed during the earlier half of the study period. An explanation for this could be the introduction of slimmer scopes during the past few years, where previously untraversable tumours can nowadays be traversed. This might introduce bias, notably selection and confounding. The latter is also a consequence of the observational nature of this study, as the participants were not randomized to a planned stoma or not. While some attempt to mitigate this was done through confounder adjustment, there is nonetheless a high risk of residual bias, probably acting unfavourably upon the planned stoma group. In addition, the small sample size only permitted adjustment for the most important confounders.

The current report focuses solely on patients initially deemed to be curable. However, the question whether to construct a pretreatment stoma could arise even when treatment intent is not curative, as chemotherapy or irradiation might be viable palliative options. Thus, future studies could potentially include or focus on palliative patients as well.

To summarize, patients with endoscopically obstructing rectal cancer, who are otherwise free of obstructive symptoms, may experience delayed treatment if given a defunctioning stoma before initiation of said treatment. Moreover, there is a pattern indicating that such a stoma could also predispose for complications before resectional surgery, although confirmatory studies are likely needed to further support this conclusion. It remains unclear whether these differences have any clinical relevance in regard to prognosis.

### Supplementary Information

Below is the link to the electronic supplementary material.

Supplementary file1 (DOCX 101 KB)

## Data Availability

Upon reasonable request, data and methodology can be shared. This also applies to the registry-based data used in the present report, while access to such data might be subject to external review by the Swedish Colorectal Cancer Registry steering committee.

## Declarations

### Permission to reproduce material from other sources

No material from other sources was included in this study.

## References

[1] Nationell kvalitetsregisterrapport ändtarmscancer 2018 (2019) Regional Oncological Centre, Umeå

[2] Fazeli MS, Keramati MR (2015). Rectal cancer: a review. Med J Islam Repub Iran.

[3] Patel JA, Fleshman JW, Hunt SR, Safar B, Birnbaum EH, Lin AY (2012). Is an elective diverting colostomy warranted in patients with an endoscopically obstructing rectal cancer before neoadjuvant chemotherapy?. Dis Colon Rectum.

[4] Smith SA, Ronksley PE, Tan Z, Dixon E, Hemmelgarn BR, Buie WD (2021). New ileostomy formation and subsequent community-onset acute and chronic kidney disease: a population-based cohort study. Ann Surg.

[5] Gavriilidis P, Azoulay D, Taflampas P (2019). Loop transverse colostomy versus loop ileostomy for defunctioning of colorectal anastomosis: a systematic review, updated conventional meta-analysis, and cumulative meta-analysis. Surg Today.

[6] Anderson BJ, Hill EG, Sweeney RE, Wahlquist AE, Marshall DT, Staveley O'Carroll KF (2015). The impact of surgical diversion before neoadjuvant therapy for rectal cancer. Am Surg.

[7] Katayama H, Kurokawa Y, Nakamura K, Ito H, Kanemitsu Y, Masuda N (2016). Extended Clavien-Dindo classification of surgical complications: Japan Clinical Oncology Group postoperative complications criteria. Surg Today.

[8] Doyle DJ, Amandeep Goyal PB, Garmon EH (2021) American Society of Anesthesiologists Classification. StatPearls 28722969

[9] Rosen RD, Sapra A (2021) TNM Classification. StatPearls 31985980

[10] Bewick V, Cheek L, Ball J (2004). Statistics review 12: survival analysis. Crit Care.

[11] Ohman U (1982). Prognosis in patients with obstructing colorectal carcinoma. Am J Surg.

