# Supplementary material for: Defunctioning stoma before neoadjuvant treatment or resection of endoscopically obstructing rectal cancer
Source: Int J Colorectal Dis. 2023 Jan 26;38(1):24. doi: 10.1007/s00384-023-04318-8 (PMC9877073; doi:10.1007/s00384-023-04318-8)

## Supplementary information

**Supp. Fig. 1** Kaplan-Meier failure curve showing survival over a 5-year period for 57 patients with endoscopically obstructing rectal cancer, stratified by a planned pretreatment stoma


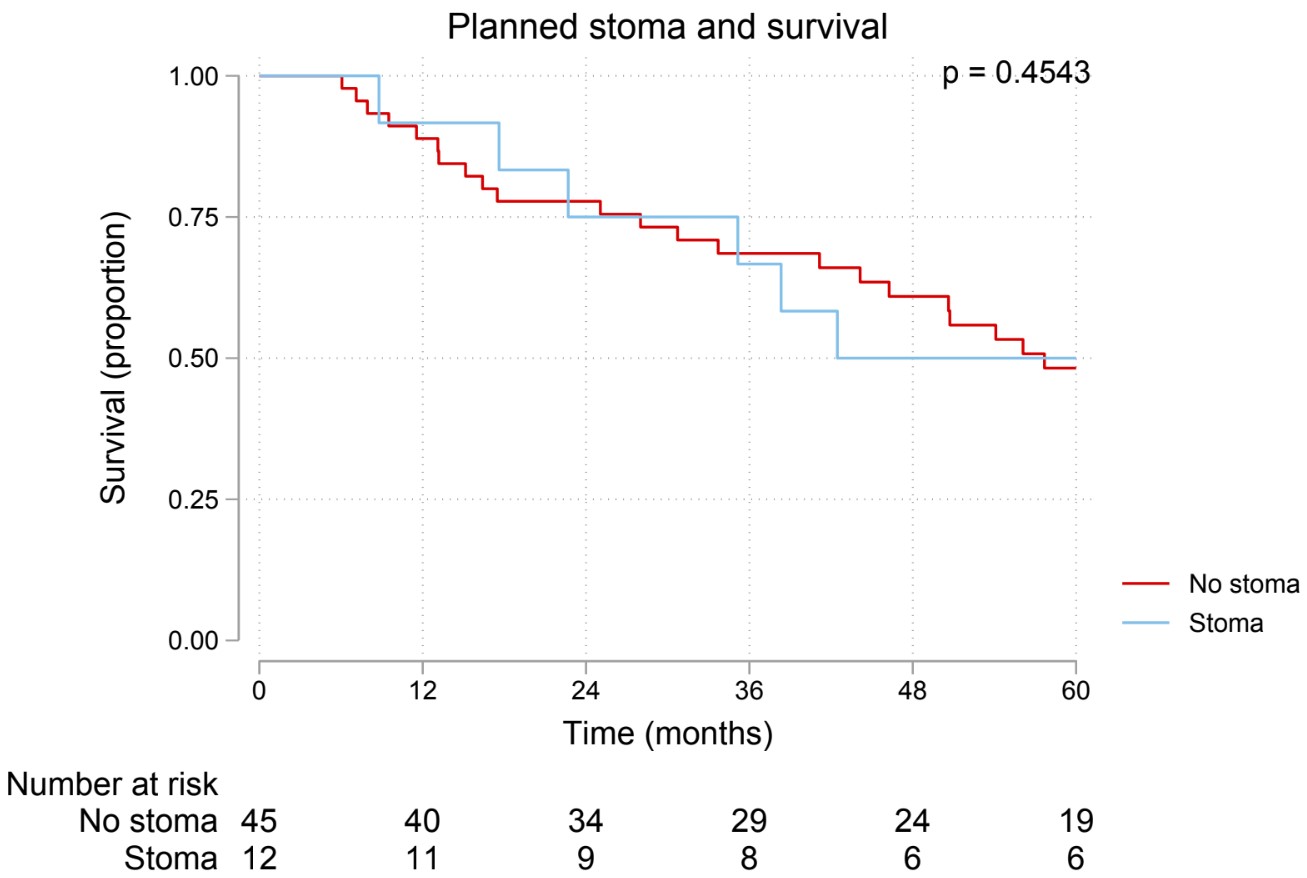

Supplement: Supplementary file 1 — Supplementary file1 (DOCX 101 KB) [file 384_2023_4318_MOESM1_ESM.docx]
